# Examination of Artificial MiRNA Mimics with Centered–Site Complementarity for Gene Targeting

**DOI:** 10.1371/journal.pone.0072062

**Published:** 2013-08-27

**Authors:** Shu-Guang Zhang, Chun-Yan Liu, Li Li, Tong-Wen Sun, Yong-Gang Luo, Wen-Jing Yun, Jin-Ying Zhang

**Affiliations:** 1 Department of MICU, the First Affiliated Hospital, Zhengzhou University, Zhengzhou, P. R. China; 2 Department of Pediatrics, the Second Affiliated Hospital, Zhengzhou University, Zhengzhou, P. R. China; 3 Department of Emergency, the First Affiliated Hospital, Zhengzhou University, Zhengzhou, P. R. China; 4 Department of Cardiology, the First Affiliated Hospital, Zhengzhou University, Zhengzhou, P. R. China; IRCCS-Policlinico San Donato, Italy

## Abstract

**Background:**

MiRNA primarily acts to repress gene expression at the post-transcriptional level through imperfect complementarity of its 5′ region to the “seed site” in the 3′ untranslated region of target mRNAs, with its “3′–supplementary site” and “center site” also playing important roles under certain circumstances. The aim of this study was to test if artificial miRNA mimics (miR-Mimics) that are designed to target the “centered sites” without “seed sites” complementarity are able to repress gene expression as natural miRNAs.

**Methods:**

We designed miR-Mimics carrying centered-site matches (CS–miR-Mimics) or seed-site matches (SS–miR-Mimics) and siRNA to two antiapoptotic genes BCL2 and AKT1. We tested the gene targeting of these constructs using real-time RT-PCR and Western blot to quantify mRNA and protein levels of BCL2 and AKT1, respectively, luciferase reporter gene assay to investigate the interaction between miR-Mimics and their target sites, and cell survival assay to study the functional outcomes of the miR-Mimics.

**Results:**

We found that CS-miR-Mimic, SS-miR-Mimic and siRNA, all down regulated the mRNA and protein levels of their cognate target BCL2 or AKT1 in a concentration-dependent manner. Luciferase reporter gene assay further confirmed the functional interactions of CS–miR-Mimic, SS-miR-Mimic and siRNA with their target sites. We then observed that the miR-Mimics and siRNAs were all able to induce cell death, as indicated by the reduced survival rate of cells.

**Conclusions:**

We have provided evidence for the feasibility of CS–miR-Mimics for post-transcriptional repression of genes, which can be designed to have reduced numbers of seed type off-target sites compared to the number of target sites from an average endogenous seed–site miRNA. CS–miR-Mimics may be a novel approach for miRNA research requiring miRNA gain-of-function.

## Introduction

With the recent advance of research into microRNAs (miRNAs), this category of endogenous small non-coding ribonucleic acids (∼22 nts in length) has rapidly emerged as one of the central players of the gene network regulating expression of an extensive repertoire of genes. Thousands of miRNAs have been identified in several organisms including humans, some of which are registered in miRBase Registry (http://www.mirbase.org/; now hosted and maintained in the Faculty of Life Sciences at the University of Manchester). Computational prediction suggests there may be even a larger number of miRNAs (∼25,000 in humans) in the mammalian genome that are to be identified [1], [2]. The high sequence conservation across metazoan species suggests strong evolutionary pressure and participation of miRNAs in essential biological processes such as cell proliferation, differentiation, apoptosis, metabolism, stress, and the forth [3]–[6]. MiRNAs are also critically involved in a variety of pathological processes including human disease, such as developmental malformations, cancer, cardiovascular disease, neuronal disorders, metabolic disturbance, and viral disease [7]–[10]. Because of the wide-spread biological effects and pathophysiological implications and their small size, miRNAs have become attractive therapeutic targets for human disease.

In the past few years, we have witnessed rapid development of many innovative techniques and methodologies pertinent to miRNA research and applications [11]–[16]. These technologies have demonstrated their efficacy and reliability in producing gain-of-function or loss-of-function of miRNAs, providing new tools for elucidating miRNA functions and opening up a new avenue for the development of new agents targeting miRNAs for therapeutic aims. These stimulating advances prompted us to propose the concept of microRNA interference (miRNAi) in 2008 [9]: Manipulating the function, stability, biogenesis or expression of miRNAs to interfere with the expression of their target protein-coding mRNAs to alter the cellular functions [10].

Mature miRNAs are double-stranded with one strand being functional that is called guide strand or major strand and the other being non-functional or minor that is called the passenger strand. MiRNAs primarily act to repress gene expression at the post-transcriptional level through imperfect complementarity of the guide strand with the 3′ untranslated region (3′UTR) of target mRNAs. The sequence specificity for target recognition by the miRNA guide strand is determined by nucleotides 2–8 of its 5′ region, referred to as the ‘‘seed site’’ [4], [5], [17]. Full complementarity of seed site is normally required for repression. Additional baseparing in other regions, preferentially at positions 13–16 from 5′–end, tremendously facilitates the action, and this region is designated as 3′–supplementary or 3′–compensatory site [18]. By natural selection, the most highly conserved region of protein-coding genes for metazoan miRNAs is this seed site [4], [19], and the next most highly conserved region corresponds to 3′–supplementary/3′–compensatory site [18]. According to Bartel and colleagues [20], the high conservation of seed site raises the potential for a single miRNA to target multiple mRNAs, as many as hundreds. On the other hand, each individual protein-coding gene may be regulated by multiple miRNAs. This implies that the actions of miRNAs are not gene specific, but sequence motif specific for they can act on all genes that carry motifs matching their seed sites. Thus, when aiming to silence a particular gene using a naturally occurring miRNA, one may actually knockdown a group of genes. This property of miRNAs creates a hurdle for thorough understanding of miRNA targeting and function. To this end, we have recently developed an approach called microRNAs Mimics or miR-Mimics [9], [10], [21]. This approach is to generate non-natural double-stranded miRNA-like RNA fragments based on the seed-site recognition. Such a RNA fragment is designed to have its 5′–end 1–8 nts fully complementary to a selected motif in the 3′UTR unique to the target gene [21]. Once introduced into cells, this RNA fragment, mimicking an endogenous miRNA, can bind specifically to its target gene at its 5′region and produce post-transcriptional repression, more specifically translational inhibition, of the gene. By comparison, miR-Mimics can be designed to have reduced numbers of seed type off-target sites compared to the number of target sites from an average endogenous miRNA [9], [10], [21].

Recent studies indicate that in addition to the more popular seed-site basepairing, some miRNAs act by centered-site complementarity [20]. “Centered sites” are a unique class of miRNA target sites that lacks both perfect seed pairing and 3′–compensatory pairing and instead has 11–12 contiguous Watson–Crick pairs to the center region of the miRNA at either nucleotides 4–15 or 5–15, without substantial pairing to either the 5′ or the 3′ ends of the miRNA. Because of a much lower conservation, centered sites provide a mechanism by which different members of the same miRNA seed family can repress distinct targets [22]; or in other words, centered-site complementarity can generate more gene-specific actions with tremendously less target genes. The discovery of centered–site complementarity for miRNA actions offers an alternative design of miR-Mimics for gene-specific targeting as a new approach of miRNA gain-of-function. The present study was motivated by this notion.

## Materials and Methods

### Cell Culture

Rat embryonic ventricular cell line (H9c2) used in this study was purchased from American Type Culture Collection (ATCC, Manassas, VA) and cultured in Dulbecco's Modified Eagle Medium (DMEM).

### Synthesis of MiRNAs and Anti-miRNA Antisense Inhibitors

**Figure 1:**
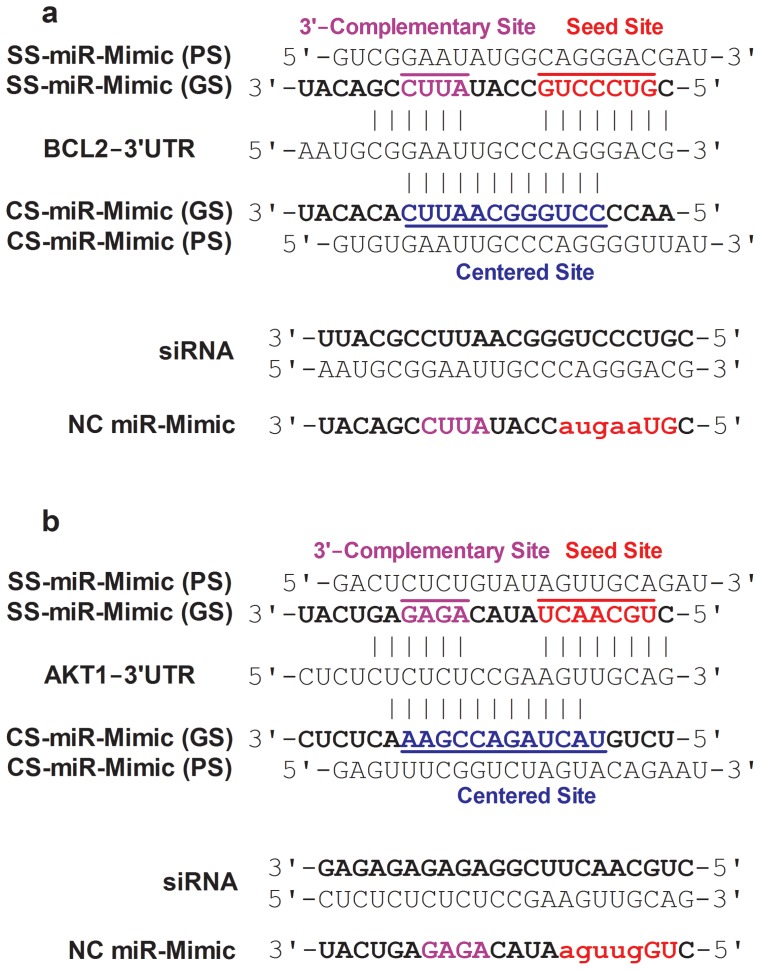
Design of centered–site miRNA mimics (CS–miR-Mimics), seed–site miRNA mimics (SS–miR-Mimics), and siRNAs targeting BCL2 (a) and AKT1 (b) genes, respectively. The centered sites (nucleotides 5–16 from 5′–end) are indicated by underlined blue letters in boldface, and the seed sites (nucleotides 2–8 at 5′–end) by boldface, red letters and the 3′–complementary sites (nucleotides 13–16 from 5′–end) by boldface, purple letters. GS: guide strand; PS: passenger strand.

MiR-Mimics, siRNAs (see Figure 1 for the sequences) and their negative control constructs were synthesized by Integrated DNA Technologies Inc (IDT). Five nucleotides or deoxynucleotides at both ends of the antisense molecules were locked (the ribose ring is constrained by a methylene bridge between the 2′-*O*- and the 4′-C atoms). Note that the sequences of target genes (BCL2 and AKT1) are highly conserved (nearly identical) across human, rat and mouse, and the gene-specific motifs used for designing miR-Mimics and siRNAs are identical among these species.

### Construction of Luciferase–MiRNA–Target Site Fusion Plasmids

To construct luciferase reporter vectors bearing miRNA-target sites, we synthesized fragments containing the exact target sites for siRNAs to BCL2 and AKT1, respectively, in the 3′UTR, through Invitrogen. These inserts were ligated into HindIII and SpeI sites in the pMIR-REPORT^TM^ luciferase miRNA expression reporter vector (Ambion) [23].

### Transfection Procedures

H9c2 cells were transfected with 1 µg of each construct with lipofectamine 2000 (Invitrogen), according to the manufacturer's instructions. Forty-eight hours after transfection, cells were used for luciferase assay or were collected for total RNA or protein purification.

### Luciferase Activity Assay

For luciferase assay involving miRNA function, H9c2 cells were transfected with the pMIR-REPORT^TM^ luciferase miRNA expression reporter vector carrying the 3′UTR of BCL2 or AKT1, as described in detail elsewhere [24].

### Quantitative Real-Time RT-PCR Analysis

The *mir*Vana™ qRT-PCR miRNA Detection Kit (Ambion) was used in conjunction with real-time PCR with TaqMan for quantification of miRNAs in our study, as previously described in detail [23], [24]. The total RNA samples were isolated with Ambion's *mir*Vana miRNA Isolation Kit, from H9c2 cells. Reactions contained *mir*Vana qRT-PCR Primer sets specific for BCL2 and AKT1, respectively. qRT-PCR was performed on a thermocycler (Mx3005PTM Realtime PCR System; Stratagene) for 40 cycles. Fold variations in expression of an mRNA between RNA samples were calculated. The threshold cycle (C_T_) is defined as the fractional cycle number at which the fluorescence passes the fixed threshold.

### Western Blot Analysis

The protein samples (membrane and cytosolic samples separately) were extracted from H9c2 cells for immunoblotting analysis, with the procedures essentially the same as described in detail elsewhere [23], [24]. The protein content was determined by BCA Protein Assay Kit using bovine serum albumin as the standard. Protein sample (∼50 µg) was fractionated by SDS-PAGE (12% polyacrylamide gels) and transferred to PVDF membrane (Millipore, Bedford, MA). The sample was incubated overnight at 4°C with the primary antibodies in 1∶200. Affinity purified rabbit polyclonal anti-bcl-2 and anti-Akt1, purchased from Cell Signaling, were used as the primary antibody. Next day, the membrane was incubated with secondary antibody (Santa Cruz Biotech, Inc.) diluted in PBS for 2 h at room temperature. Finally, the membrane was rinsed with PBS before scanning using the Infrared Imaging System (LI-COR Biosciences). GAPDH was used as an internal control for equal input of protein samples, using anti-GAPDH antibody GAPDH monoclonal antibody (Fitzgerald Industries International Inc). Western blot bands were quantified using QuantityOne software by measuring the band intensity (Area x OD) for each group and normalizing to GAPDH. The final results are expressed as fold changes by normalizing the data to the control values.

### MTT Assay for Cell Proliferation

The WST-1 kit (Roche, Penzberg, Germany). In brief, 24 h after treatment with varying constructs, H9c2 cells were washed with PBS and grown in 100 µl of fresh culture medium plus 10 µl of WST-1 reagent for 30 min. The absorbance was measured at 425 nm using a Spectra Rainbow microplate reader (Tecan, Grödig, Austria) with a reference wavelength of 690 nm [23], [25], [26].

### Data Analysis

Group data are expressed as mean ± SEM. Comparisons between groups were performed by unpaired Student's *t*-test. A two-tailed *p*<0.05 was taken to indicate a statistically significant difference.

## Results

### Design of Centered–Site miR-Mimics

We first used siRNA-prediction website BLOCK-iT™ RNAi Designer provided by Invitrogen (https://rnaidesigner.invitrogen.com/rnaiexpress/) to obtain two lists of top 10 siRNA sequences: one for BCL2 and the other for AKT1, both of which are known anti-apoptotic genes. These siRNAs were designed to target the 3′UTR of BCL2 or AKT1 mRNA. We then selected one sequence from each of the two lists that we considered to represent the optimal siRNA in terms of the gene specificity and GC content (around 55%). Based on the selected sequences, we designed centered-site miR-Mimic (CS-miR-Mimic) sequences of 22–nts in length. The guide strand (GS) of a CS-miR-Mimic contains a stretch of 12-nts at positions 5–16 from 5′–end that are contiguously complementary to a unique motif in 3′UTRs of BCL2 (**Fig.** **1a**) or AKT1 (**Fig.** **1b**) as the centered site [20], and unmatched regions at positions 1–3 and 16–22 flanking the centered site with “AU” at most 3′–end as an overhand. The 2–3 nts at the 5′end of the CS-miR-Mimics were carefully chosen to minimize the probability of creating 2–8 seed-site matches to any non-target genes. Specifically, the 2–8 nts were ACCCCUG for BCL2 and CUGUACU for AKT1. The passenger strand (PS) was exactly complementary to the guide strand except for the 3′–end “AU” overhand.

For comparison, seed-site miR-Mimics (SS–miR-Mimics) were also studied. We have previously tested SS–miR-Mimics with full seed–site matches plus 5∼6 nts contiguous basepairing at the 3′–end of the selected targets [23]. According to the conservation analysis and array data, it is now known that seed–site targets prefer to acquire supplemental pairing at positions 13–16 rather than extending pairing through nucleotides 9–12 [18]. Based on this note, we modified our original design of SS-miR-Mimics. In this study, each SS-miR-Mimic is 22-nts long, carrying first 8 nucleotides at 5′–end that are contiguously Watson–Crick pairing to 3′UTR of the test target BCL2 (**Fig.** **1a**) or AKT1 (**Fig.** **1b**) mRNA and 6 matched nucleotides covering the 3′supplementary/complementary site (positions 13–16).

A negative control miR-Mimic (NC miR-Mimic) for verifying the specificity of effects of the miR-Mimics and siRNAs was designed based on the sequence of the SS-miR-Mimics. We modified the SS-miR-Mimic sequences to contain 5 mismatched nucleotides at positions 4–8 from the 5′–end. Such a modification is expected to disrupt both the seed–site and the centered–site complementarity and render loss of the ability to bind the target mRNA with sufficient affinity to elicit repressive effects.

### Validation of Centered–Site miR-Mimics

**Figure 2:**
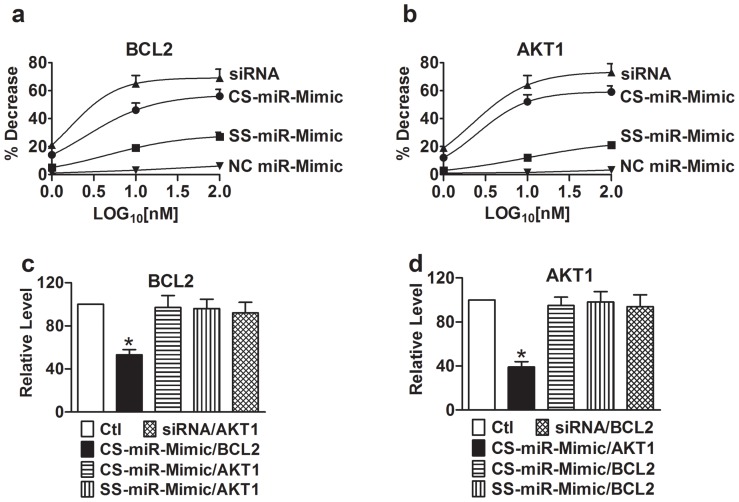
Effects of miR-Mimics on the mRNA levels of their target genes BCL2 and AKT1 in H9c2 rat ventricular cells, determined by real-time quantitative RT-PCR. (**a**) and (**b**) Concentration-response curves of BCL2 and AKT1, respectively. Measurements were made 24 hrs after transfection of cells with CS–miR-Mimics (centered site miR-Mimics), SS–miR-Mimics (seed site miR-Mimics), siRNA, or NC miR-Mimic (negative control miR-Mimic) using lipofectamine 2000. The concentrations of the constructs tested were 1, 10, and 100 nM, expressed in log10 scale. Control (Ctl) cells were mock-treated. Symbols are averaged experimental data and the curves are fits by Hill equation. For BCL2, EC_50_ = 2.5 nM for CS–miR-Mimic, EC_50_ = 4.6 nM for SS-miR-Mimic, and EC_50_ = 1.6 nM for siRNA. For AKT1, EC_50_ = 2.5 nM for CS–miR-Mimic, EC_50_ = 10 nM for SS-miR-Mimic, and EC_50_ = 2.1 nM for siRNA. Note that the constructs for AKT1 failed to affect BCL2 (**c**) and the constructs for BCL2 failed to affect AKT1 (**d**). *p<0.05 *vs*. Ctl; n = 5 for each group.

We measured the mRNA levels of BCL2 (**Fig.** **2a**) and AKT1 (**Fig.** **2b**) using qPCR methods in H9c2 cells after treatment with the constructs by transfection. Our data showed that all constructs, CS-miR-Mimic, SS-miR-Mimic, and siRNA, negatively regulated their cognate target BCL2 or AKT1 in a concentration-dependent manner (1, 10 and 100 nM). But the efficacy was different among the constructs in an order of siRNA > CS–miR-Mimic > SS–miR-Mimic. No cross-effects on BCL2 (**Fig.** **2c** **)** and AKT1 (**Fig.** **2d** **)** were observed. Furthermore, NC miR-Mimic produced minimal effects.

**Figure 3:**
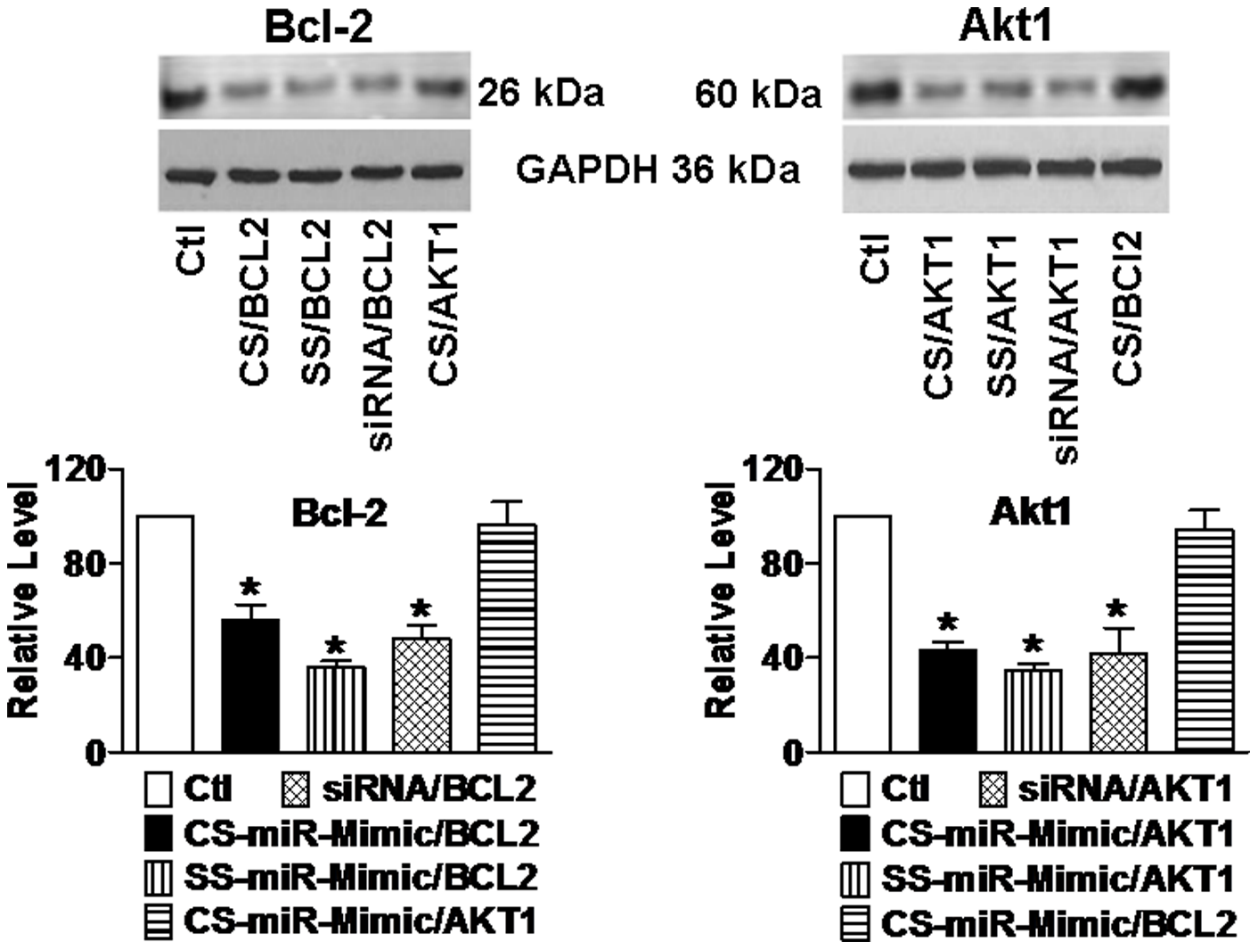
Effects of miR-Mimics on the protein levels of Bcl-2 (a) and Akt1 (b) in H9c2 rat ventricular cells, determined by Western blot analysis. Upper panels: representative immunoblotting bands; lower panels: averaged band densities. Measurements were made 24 hrs after transfection of cells with varying constructs using lipofectamine 2000. CS/BCL2 = CS-miR-Mimic/BCL2 targeting BCL2; CS/AKT1 = CS–miR-Mimic/AKT1 targeting AKT1. The concentrations of the constructs tested were 10 nM. Control (Ctl) cells were mock-treated. *p<0.05 *vs*. Ctl; n = 4 for each group.

Consistent with the mRNA data, our subsequent Western blot analysis demonstrated significant downregulation of protein levels of Bcl-2 (**Fig.** **3, left panel** **s**) and Akt1 (**Fig.** **3, right panel** **s**) by all three constructs. The results indicate that the gene silencing effects of the CS–miR-Mimics reached a functional level. NC miR-Mimic failed to elicit any appreciable effects.

**Figure 4:**
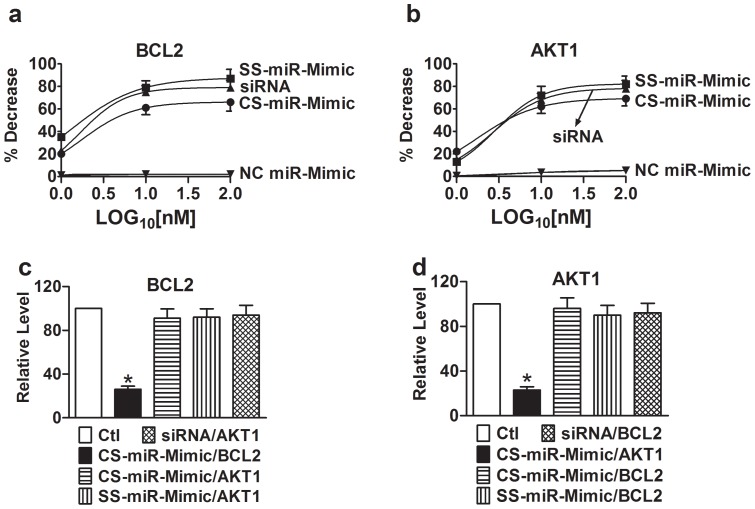
Interactions between miR-Mimics and their target sites in, as reported by luciferase activity assay with the pMIR-REPORT^TM^ luciferase miRNA expression reporter vector carrying the BCL2 or AKT1 3′UTR in H9c2 rat ventricular cells. (a) and (b) Concentration-response curves. Measurements were made 24 hrs after transfection of cells with varying constructs using lipofectamine 2000. The concentrations of the constructs tested were 1, 10, and 100 nM, expressed in log10 scale. Control (Ctl) cells were transfected with the luciferase vector alone without miR-Mimics. Symbols are averaged experimental data and the curves are fits by Hill equation. For BCL2, EC_50_ = 1.7 nM for CS-miR-Mimic, EC_50_ = 1.3 nM for SS-miR-Mimic, and EC_50_ = 1.7 nM for siRNA. For AKT1, EC_50_ = 1.7 nM for CS-miR-Mimic, EC_50_ = 2.9 nM for SS-miR-Mimic, and EC_50_ = 2.7 nM for siRNA. Note that the constructs for AKT1 failed to affect BCL2 (c) and the constructs for BCL2 failed to affect AKT1 (d). *p<0.05 *vs*. Ctl; n = 4 for each group.

To verify the above results, we went on to conduct luciferase reporter gene assay. As illustrated in Figure 4, the CS-miR-Mimics substantially reduced the luciferase activities elicited by the vectors carrying their respective target sites at 3′UTR of the luciferase gene, but had no significant effects when co-transfected with the vectors that do not contain their target sites. For instance the BCL2 CS-miR-Mimic minimally affected AKT1 target site (**Fig.** **4c**); *vice versa*, the AKT1 CS-miR-Mimic failed to alter the luciferase activity with BCL2 target site. As comparisons, the SS-miR-Mimics and siRNAs were also able to suppress the luciferase activities with the corresponding target sites.

**Figure 5:**
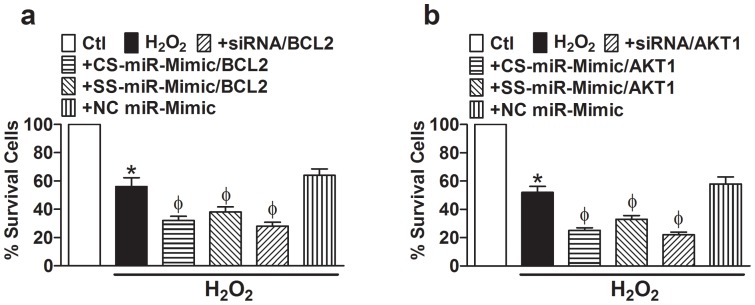
Effects of various miR-Mimics targeting BCL2 (a) or AKT1 (b) on H9c2 cell survival evaluated with MTT assay. Cell death was induced by incubating with H_2_O_2_ (50 µM). “+” indicates H_2_O_2_ + construct. *p<0.05 *vs*. Ctl; ^Φ^ *p*<0.05 *vs*. H_2_O_2_ alone; n = 5 for each group.

We then continued to investigate the effects of the two CS–miR-Mimics on the cell death induced by oxidative stress. As depicted in Figure 5, incubation of cells with H_2_O_2_ induced substantial cell death (∼45%), as indicated by the reduced cell survival, and addition of CS–miR-Mimic to BCL2 (**Fig.** **5a**) or to AKT1 (**Fig.** **5b**) significantly exacerbated the cell death (increased to ∼70%). Similar effects were seen with the SS–miR-Mimics (∼60% cell death) and siRNAs (∼75% cell death), but not with the NC miR-Mimic.

## Discussion

In the present study, we provided evidence for the feasibility of centered–site artificial miR-Mimics for post-transcriptional repression of genes with improved gene-specificity of actions. We tested targeting of two CS–miR-Mimics on two selected genes BCL2 and AKT1, as compared with the actions of seed–site miR-Mimics and siRNAs. The targeting was validated from several aspects including expression regulation by measurements of mRNAs and proteins, miR-Mimic–target interactions by measurements of luciferase reporter gene activities, and functional outcome by measurements of cell death/survival.

The targeting at the mRNA level revealed differential efficacies and potencies among the constructs with siRNA showing the largest silencing effects, followed by CS–miR-Mimic and then SS–miR-Mimic. These differences can be explained by the fact that siRNA fully base-pairs target genes, whereas CS–miR-Mimic and SS-miR-Mimic have only partial target complementarity. CS–miR-Mimic covers the cleavage site (nucleotides 11–12 from 5′–end) that mediate Ago-catalyzed cleavage [20], [26], [27] but SS-miR-Mimic does not; thus, CS–miR-Mimic is expected to produce larger repressive effects than SS-miR-Mimic. The differences were minimized with luciferase assay. This is likely because data from luciferase assay involve changes of both mRNA and protein and SS–miR-Mimic primarily exerts inhibition of protein translation in addition to destabilizing mRNA. This property of SS–miR-Mimic enables it to produce greater effects at the functional level compared with its effects at the mRNA level.

Gain-of-function is an indispensible approach in miRNA research and may be in disease therapy as well for downregulated miRNAs. A common strategy to achieve gain-of-function is to introduce synthetic canonical miRNAs into cells by means of transfection using lipid carriers and of infection using viral vectors. This approach is virtually the gene replacement therapy. By comparison, miR-Mimics are non-natural artificial nucleotide fragments that act as endogenous miRNAs. In addition to this major distinction, miR-Mimics differ from natural miRNAs (including exogenously supplied synthetic canonical miRNAs) in that they are designed to interact with sequence motifs at 3′UTRs unique to the target genes; therefore unlike a natural miRNA that may have hundreds of targets, each miR-Mimic theoretically has less target genes. Thus, the action of miR-Mimic may have improved gene specificity while that of miRNAs are not gene specific. In other words, a miR-Mimic can act only on its particular target gene, but a native miRNA can act on any genes that carry its binding sequence. From these points of view, miR-Mimics bear a resemblance to siRNAs that are also non-natural artificial constructs acting in a gene-specific manner [28], [29]. However, miR-Mimics are different from siRNAs in that they are partially complementary to targets and thus act by miRNA mechanisms. SS–miR-Mimics have only moderate effects on target stability whereas siRNAs mainly act to degrade targets; this is also demonstrated in the present study (**Fig.** **2**). Moreover, miR-Mimics are designed according to the sequence in the 3′UTR of target mRNAs while siRNAs can be designed in any regions of selected target genes.

The seed–site match is a source of multiple-target property of miRNA actions because of the high conservation of seed sequence across protein-coding genes. The seed–site match is also a reason for the off-target effects of siRNAs [28]–[30]. CS–miR-Mimics are designed to have reduced numbers of seed–site match that generates off-target effects compared to the number of target sites from an average endogenous seed–site miRNA, and are thus expected to have relatively smaller odds of non-gene-specific actions. Because of the flexibility of designing the 5′-end 1–3 nts of a CS-miR-Mimic, one can place selected nucleotides in these three positions to minimize the probability of seed-site (5′-end 2–8 nts) complementarity to non-target genes. By comparison, the designing of siRNAs does not offer this advantage. In this sense, this feature may be an advantage of CS–miR-Mimics over miRNAs and siRNAs and even SS–miR-Mimics as well. This issue however needs to be experimentally verified by future studies.

## Conclusion

The miR-Mimic technology utilizing synthetic, non-natural nucleic acids that can bind to the unique sequence of target genes (mRNAs) and elicit post-transcriptional repressive effects as an endogenous miRNA does. A fundamental requirement to be satisfied for the design of miR-Mimics is that the 3′UTR of the target gene must contain a unique sequence distinct from other genes to enhance gene-specific action. We report here the design and experimental validation of CS–miR-Mimics. The results indicate that the miR-Mimic approach can be diversified into CS–miR-Mimics and SS-miR-Mimics, and CS–miR-Mimics might be a better choice when considering the possible better gene specificity than SS–miR-Mimics. CS–miR-Mimics may be used as a new tool for miRNA research requiring miRNA gain-of-function and for therapeutic purpose on conditions associated with miRNA deregulation.
